# Effective Pain Relief Following Caudal Epidural Block in a Pediatric Patient with Traumatic Sacrococcygeal Dislocation: A Case Report

**DOI:** 10.3390/children13010033

**Published:** 2025-12-25

**Authors:** Jeongsoo Choi, Da Hyung Kim, Jin Hun Chung, Ho Soon Jung, Yong Han Seo, Hea Rim Chun, Hyung Yoon Gong, Jae Young Ji, Jin Soo Park, Jun Yong Jeong, Sohyeon Ka

**Affiliations:** Department of Anesthesiology and Pain Medicine, Soonchunhyang University Hospital Cheonan, Cheonan 31151, Republic of Korea; 124393@schmc.ac.kr (J.C.); 121826@schmc.ac.kr (D.H.K.); anesth70@schmc.ac.kr (J.H.C.); c75501@schmc.ac.kr (Y.H.S.); blau00@schmc.ac.kr (H.R.C.); 83466@schmc.ac.kr (H.Y.G.); 118541@schmc.ac.kr (J.S.P.); 138443@schmc.ac.kr (J.Y.J.); 144120@schmc.ac.kr (S.K.)

**Keywords:** pediatric trauma, sacrococcygeal dislocation, coccydynia, caudal epidural block, regional anesthesia

## Abstract

***Background and* *Clinical Significance:*** Sacrococcygeal joint dislocation is an extremely rare traumatic condition in the pediatric population and is typically caused by direct trauma to the gluteal region. Most reported cases have been managed conservatively with analgesics or manual reduction, and the application of a caudal epidural block in children with this entity has, to our knowledge, never been previously described. ***Case Presentation:*** A 14-year-old girl presented with aggravated coccydynia following a second fall. Six months earlier, she had been diagnosed with sacrococcygeal dislocation after her initial fall, and her symptoms had been well controlled at a Numerical Rating Scale (NRS) score of 3 with acetaminophen and nonsteroidal anti-inflammatory drugs. However, after the recent reinjury, her pain worsened to an NRS score of 6 and did not improve despite continued conservative pharmacologic treatment. Radiographic examination at our institution confirmed anterior angular displacement of the coccyx. Accordingly, an ultrasound-guided caudal epidural block was performed using mepivacaine and dexamethasone. At follow-up evaluations conducted 2 weeks and 2 months after the procedure, her pain had decreased to an NRS score of 2, accompanied by functional improvement. ***Conclusions:*** This case suggests that caudal epidural block may serve as a safe and potentially effective therapeutic option for pediatric patients experiencing coccygeal pain following traumatic sacrococcygeal joint dislocation.

## 1. Introduction

Sacrococcygeal dislocation, in which the coccyx becomes separated from the sacrum and is displaced either anteriorly or posteriorly, is rarely reported in pediatric patients [1]. Sacrococcygeal dislocation is primarily caused by trauma, and anterior dislocation is more common than posterior dislocation [2]. The resulting coccydynia refers to pain arising from the coccygeal region, and because no standardized treatment has been established, the condition may progress to chronic pain. In female patients, this condition warrants particular attention, as childbirth is a recognized cause of coccygeal injury, including sacrococcygeal dislocation, which may further exacerbate or perpetuate coccygeal pain [3]. In addition, in female patients, it may pose potential complications during labor. Treatment options include physical therapy, nonsteroidal anti-inflammatory drugs (NSAIDs), steroid injections into the joint, manual reduction, surgical fixation of the sacrum and coccyx, and coccygectomy [4,5,6]. Caudal epidural block is widely used for managing lower sacral and coccygeal pain, providing both anti-inflammatory effects and modulation of sympathetic activity. Although caudal block is considered a safe procedure in pediatric patients, its use for alleviating pain associated with sacrococcygeal dislocation is rarely reported. We describe the case of a 14-year-old girl with persistent coccydynia following sacrococcygeal dislocation whose symptoms improved after a caudal epidural block. This case highlights the potential utility of caudal epidural block as a safe and effective interventional option for refractory coccygeal pain in pediatric patients.

## 2. Case Presentation

**Figure 1:**
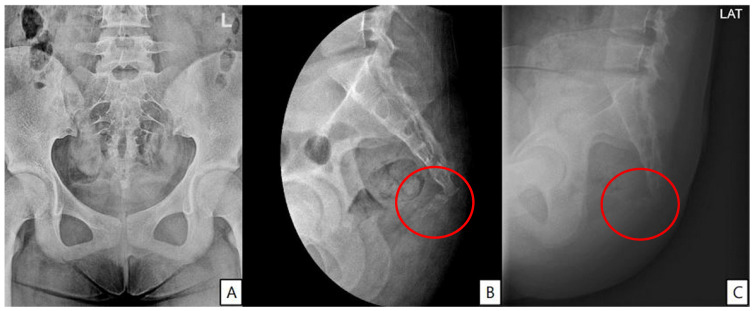
(**A**) Anteroposterior pelvic radiograph showing no visible fracture but overall coccygeal axis deviation. (**B**) Dynamic lateral view showing anterior angulation of the coccyx at the sacrococcygeal junction (red circle). (**C**) Seated lateral view demonstrating accentuated anterior displacement, suggestive of coccygeal instability (red circle).

**Figure 2:**
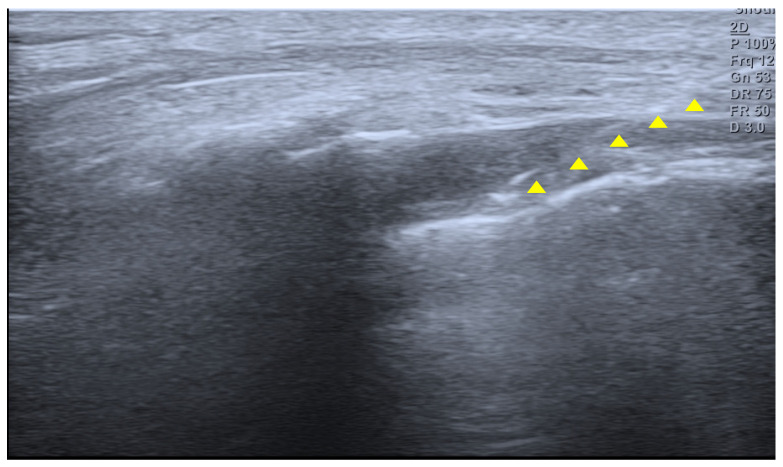
Ultrasound image of the sacral hiatus during caudal epidural block. The needle is seen advancing through the sacrococcygeal ligament into the caudal epidural space under real-time guidance (yellow triangle indicates the needle).

A 14-year-old girl developed coccygeal pain following a fall inside a bus six months earlier. At that time, she was diagnosed with sacrococcygeal dislocation at an outside hospital and was prescribed acetaminophen and NSAIDs, which maintained her symptoms at a Numerical Rating Scale (NRS) score of approximately 3. However, one month prior to presentation, she sustained a second fall to the same area, after which her pain worsened despite continued pharmacologic treatment, reaching an NRS score of 6. She was therefore referred to our pain clinic. The radiographs obtained at the time of the initial injury were unavailable. Radiography performed at our institution demonstrated anterior angulation of the coccyx (Figure 1). Physical examination revealed tenderness over the sacrum and coccyx. The patient was able to sit but could not remain seated for prolonged periods due to pain and reported worsening pain upon standing. No cutaneous abnormalities or swelling were noted over the coccygeal region. She denied defecation-related pain. For pain control, an ultrasound-guided caudal epidural block was performed (Figure 2). Prior to the procedure, the patient and guardians received a detailed explanation of the block. As the patient was cooperative and fully understood the process, the procedure was performed without sedation or anxiolysis, using verbal reassurance alone. Under sterile conditions, the patient was positioned prone, and the sacral hiatus was identified using ultrasound. After local anesthesia of the skin at the needle insertion site, a butterfly catheter was advanced toward the sacral hiatus under an out-of-plane view, traversing the sacrococcygeal ligament. The needle was then redirected in-plane into the epidural space. A mixture of 1 mL of 2% mepivacaine, 8 mL of 0.9% normal saline, and 5 mg of dexamethasone was injected. At the 2-week follow-up, although the qualitative character of pain remained similar, the patient reported an increased ability to remain seated and a reduced frequency of pain when standing. Her pain intensity decreased significantly to an NRS score of 2, accompanied by noticeable functional improvement. At the 2-month follow-up, her pain remained at an NRS score of 2, and she maintained a level of daily functioning without significant limitations.

## 3. Discussion

Sacrococcygeal dislocation is a rare injury in pediatric patients, as evidenced by population-based studies in which it is categorized among the least common types of traumatic joint dislocations [7,8]. Only a small number of cases have been reported in the literature to date [1,2,7]. Most cases are known to result from falls or direct trauma to the buttock region [2]. The sacrum and coccyx are connected at the sacrococcygeal joint, an amphiarthrodial articulation that allows limited flexion and extension. This joint is stabilized by the anterior and posterior sacrococcygeal ligaments, the lateral sacrococcygeal ligaments, and the anococcygeal raphe. Surrounding pelvic floor musculature, including the levator ani and the gluteus maximus, also contributes to regional support. Sacrococcygeal dislocation refers to malalignment or marked anterior angulation of the articulation between the sacrum and coccyx, which can be identified on radiographic evaluation. In particular, dynamic lateral radiographs define pathologic instability when the coccygeal flexion angle exceeds 25°, or when the coccyx demonstrates more than 25% anterior translation relative to its normal position [9]. Such dislocation can cause pain when it results in injury to the sacrococcygeal joint and its supporting ligaments.

**Figure 3:**
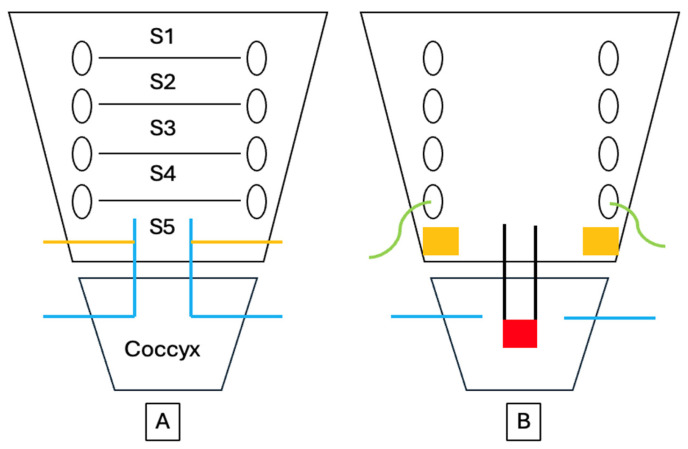
Schematic diagram of the nerve innervation of the sacrococcygeal region. Panel (**A**) shows the anterior view and panel (**B**) the posterior view. The coccygeal nerve (Co1) is indicated in blue, the S5 nerve in yellow, the S4 branch in green, the sympathetic chain in black, and the ganglion impar in red.

The pain that develops after sacrococcygeal dislocation is primarily somatic in origin and arises from injury to the periosteum, joint capsule, and surrounding ligamentous structures. This region is innervated by the coccygeal plexus, a small plexus formed by the ventral rami of the sacral nerves S4 and S5 and the coccygeal nerve Co1 [10] (Figure 3). The anococcygeal nerve, which branches from this plexus, provides sensory innervation to the sacrococcygeal junction and the skin over the coccygeal tip. Additional innervation arises from terminal branches of the sacral nerves and sympathetic fibers originating from the inferior hypogastric sympathetic chain. Consequently, nociceptive stimuli from the dislocated sacrococcygeal region are transmitted primarily through the S4–Co1 nerve roots, and patients typically experience exacerbation of pain when sitting on a hard surface or rising from a seated position. In particular, the abrupt pain that occurs when rising from a seated position is recognized as a characteristic symptom of coccygeal instability. Maigne et al. [11] reported that this positional pain is a specific finding observed only in patients with coccygeal dislocation or hypermobility [12]. Anterior displacement or shearing forces applied to the coccyx during trauma can directly stimulate nociceptors, producing pain and referred symptoms through both somatic and sympathetic pathways. Consequently, the exacerbation of pain when sitting or rising from a seated position after sacrococcygeal dislocation can be understood as the result of this complex interplay of nociceptive transmission mechanisms.

Caudal epidural block is an effective procedure for managing coccygeal pain as well as lower lumbar and sacral discomfort, and it is one of the most performed nerve blocks in pediatric patients. The technique involves injecting local anesthetics into the sacral epidural space, thereby blocking sensory and sympathetic fibers, including the sacral and coccygeal nerve roots. It provides effective relief of somatic pain originating from the periosteum and joint capsule, and depending on the volume administered, the injectate may spread to the level of the ganglion impar, offering potential suppression of visceral pain [13]. In young children, the anatomical characteristics of the sacrum facilitate the performance of this technique. The sacral hiatus is relatively wide and superficial, and distance between hiatus and termination of dural sac provides an adequate safety margin for needle advancement. In addition, the depth of epidural space is only minimally influenced by age or body weight in early childhood, contributing to a high success rate. Caudal epidural block is commonly indicated for procedures involving the lower abdomen, pelvis, and lower extremities. Major sacral malformations, central nervous system infection, and intracranial hypertension constitute contraindications. The reported failure rate of caudal epidural block ranges from approximately 5% to 10%. The use of ultrasound has been shown to improve success rates. However, outcomes remain largely dependent on operator experience and anatomical variation of the sacral hiatus [14]. Nevertheless, compared with other neuraxial techniques, caudal epidural block is generally regarded as having a relatively short learning curve due to the superficial location of the sacral hiatus and the reproducibility of anatomical landmarks, particularly when ultrasound guidance is used [15]. With growth, narrowing or closure of the sacral hiatus and increased epidural fat density may reduce the spread of local anesthetics, making the technique less suitable in older children. In the present case, the patient was 14 years old, and alternative interventional options such as ganglion impar block could have been considered. However, ganglion impar block often requires fluoroscopic guidance and procedural sedation which may increase procedural complexity and invasiveness in pediatric patients. Therefore, caudal epidural block was selected as a less invasive and more familiar technique, allowing effective coverage of sacral nerve roots without the need for additional imaging. To assess the therapeutic response while minimizing prolonged sensory blockade, 2% mepivacaine which has a rapid onset and intermediate duration of action, was chosen and combined with dexamethasone. When corticosteroids are added, the block can further attenuate local inflammatory responses by reducing the release of inflammatory mediators such as prostaglandins, substance P, and pro-inflammatory cytokines (e.g., IL-6), thereby mitigating peripheral sensitization. Thus, caudal epidural block relieves coccygeal pain both by interrupting somatic nociceptive transmission and by diminishing sympathetic-mediated pain pathways. Ganglion impar block is typically used in patients with chronic coccydynia [16], targeting the sympathetic ganglion located anterior to the coccyx with local anesthetic to achieve pain relief. In contrast, caudal epidural block provides a broader approach. Although it may be particularly effective for pain with an inflammatory component, its blockade of sacral nerve roots and potential spread to the sympathetic ganglion can yield pain-relieving effects comparable to those of a ganglion impar block [17].

Previous reports of pediatric sacrococcygeal dislocation have described symptom improvement with conservative management, such as pharmacologic treatment or manual reduction [1,7]. In the present case, the patient initially responded to medication; however, after sustaining an additional traumatic event, her pain worsened, and meaningful improvement could no longer be expected with pharmacologic therapy alone. Because the sacrococcygeal joint remains anatomically immature in growing children, sacrococcygeal dislocation may resemble an injury involving the growth plate, as suggested in previous studies. In such situations, reduction maneuvers or more invasive interventions should be approached with caution, as they may increase the risk of further injury or secondary complications. Hamoud et al. [1] reported that coccygeal fracture–dislocation in younger patients often improves effectively with conservative treatment alone, while manual reduction shows a high failure rate and is therefore not recommended. Considering these factors, we selected caudal epidural block as a relatively safe intervention with the potential to provide therapeutic benefit. Following the procedure, the patient exhibited notable pain relief and functional improvement. While this temporal association suggests that the caudal epidural block may have contributed to the observed clinical benefits, it is important to acknowledge that pediatric patients possess a substantial capacity for spontaneous recovery. Therefore, the possibility that these improvements reflect, at least in part, the natural course of healing cannot be excluded. This report has several limitations. The absence of a control group precludes definitive attribution of the therapeutic response to the intervention itself. Additionally, the relatively short follow-up period and the complex interplay between radiographic abnormalities and symptomatology make it challenging to elucidate the precise mechanism underlying the clinical improvement.

## 4. Conclusions

In pediatric patients with coccygeal pain following traumatic sacrococcygeal dislocation, caudal epidural block may be considered a potential therapeutic option. However, given the inherent limitations of a single case report and the capacity for spontaneous recovery in this population, further studies involving larger cohorts and extended follow-up are needed to clarify the efficacy and long-term outcomes of this approach.

## Data Availability

Data is contained within the article.

## Abbreviations

NRS	Numerical Rating Scale
NSAIDs	Nonsteroidal anti-inflammatory drugs

## References

[1] Hamoud K., Abbas J. (2015). Fracture dislocation of the sacro-coccygeal joint in a 12-year-old boy. A case report and literature review. Orthop. Traumatol. Surg. Res..

[2] Panigrahi V.P., Adsul N., Chahal R.S., Kalra K.L., Acharya S. (2020). Traumatic posterior dislocation of sacrococcygeal joint: A case report and review of the literature. Surg. Neurol. Int..

[3] Márquez-Carrasco Á.M., García-García E., Aragúndez-Marcos M.P. (2019). Coccyx pain in women after childbirth. Enfermería Clínica (Engl. Ed.).

[4] Maigne J.Y., Chatellier G., Faou M.L., Archambeau M. (2006). The treatment of chronic coccydynia with intrarectal manipulation: A randomized controlled study. Spine (1976).

[5] Bergkamp A.B., Verhaar J.A. (1995). Dislocation of the coccyx: A case report. J. Bone Joint Surg. Br..

[6] Kim W.Y., Han C.W., Kim Y.H. (2004). Joystick reduction and percutaneous pinning for an acutely anteriorly dislocated coccyx: A case report. J. Orthop. Trauma.

[7] Kanabur P., Gowd A., Bulkeley J.A., Behrend C.J., Carmouche J.J. (2017). Symptomatic sacrococcygeal joint dislocation treated using closed manual reduction: A case report with 36-month follow-up and review of literature. Trauma Case Rep..

[8] Söderling W.J., Helenius I.J., Grahn P.M., Gissler M.V., Laaksonen T.A., Ahonen M.M. (2025). Population-based incidence and demographic patterns of traumatic joint dislocations in children and adolescents. Acta Orthop..

[9] Maigne J.Y., Tamalet B. (1996). Standardized radiologic protocol for the study of common coccygodynia and characteristics of the lesions observed in the sitting position. Clinical elements differentiating luxation, hypermobility, and normal mobility. Spine (1976).

[10] Woon J.T., Stringer M.D. (2012). Clinical anatomy of the coccyx: A systematic review. Clin. Anat..

[11] Maigne J.Y., Doursounian L., Chatellier G. (2000). Causes and mechanisms of common coccydynia: Role of body mass index and coccygeal trauma. Spine (1976).

[12] Maigne J.Y., Guedj S., Fautrel B. (1992). Coccygodynia: Value of dynamic lateral x-ray films in sitting position. Rev. Rhum. Mal. Osteoartic..

[13] Lundblad M., Eksborg S., Lonnqvist P.A. (2012). Secondary spread of caudal block as assessed by ultrasonography. Br. J. Anaesth..

[14] Liu J., Wu X., Li R., Zhang Y. (2012). A comparison of ultrasonography versus traditional approach for caudal block in children. Zhonghua Yi Xue Za Zhi.

[15] Wiegele M., Marhofer P., Lönnqvist P.-A. (2019). Caudal epidural blocks in paediatric patients: A review and practical considerations. Br. J. Anaesth..

[16] Gonnade N., Mehta N., Khera P.S., Kumar D., Rajagopal R., Sharma P.K. (2017). Ganglion impar block in patients with chronic coccydynia. Indian J. Radiol. Imaging.

[17] Sencan S., Yolcu G., Bilim S., Kenis-Coskun O., Gunduz O.H. (2022). Comparison of treatment outcomes in chronic coccygodynia patients treated with ganglion impar blockade versus caudal epidural steroid injection: A prospective randomized comparison study. Korean J. Pain.

